# Historical global ocean wave data simulated with CMIP6 anthropogenic and natural forcings

**DOI:** 10.1038/s41597-023-02228-6

**Published:** 2023-05-26

**Authors:** Anindita Patra, Guillaume Dodet, Mickaël Accensi

**Affiliations:** Univ Brest, CNRS, Ifremer, IRD, Laboratoire d’Océanographie Physique et Spatiale (LOPS), 29280 Plouzané, France

**Keywords:** Physical oceanography, Physical oceanography

## Abstract

This dataset presents historical ocean wave climate during 1960–2020, simulated using the numerical model WAVEWATCH III (WW3) forced by Coupled Model Intercomparison Project phase 6 (CMIP6) simulations corresponding to natural-only (NAT), greenhouse gas-only (GHG), aerosol-only (AER) forcings, combined forcing (natural and anthropogenic; ALL), and pre-industrial control conditions. Surface wind at 3-hourly temporal resolution, and sea-ice area fraction at monthly frequency, from a CMIP6 model - MRI-ESM2.0 are used to force WW3 over the global ocean. Model calibration and validation of the significant wave height are carried out using inter-calibrated multi-mission altimeter data produced by the European Space Agency Climate Change Initiative, with additional corroboration using ERA-5 reanalysis. The simulated dataset is assessed for its skill to represent mean state, extremes, trends, seasonal cycle, time consistency, and spatial distribution over time. Numerically simulated wave parameters for different individual external forcing scenario is not available yet. This study produces a novel database particularly useful for detection and attribution analysis to quantify the relative contributions of natural and anthropogenic forcings to historical changes.

## Background & Summary

The relevance of surface ocean wind waves is manifold, from environmental to socioeconomic impacts on human society. A wide range of applications of wave information already stated by many previous literature include ship routing^1^, coastal and offshore engineering activities^2^, assessment of coastal hazards^3^, wave energy extraction planning^4^, estimation of ocean–atmosphere exchange^5^, among others. The importance of dynamic coupling of wave processes into coupled ocean–atmosphere global climate models has been emphasized since long back as surface wind-waves are capable of directly affecting climate system through continuous ocean-atmosphere exchange^5^.

Despite being a key component of the climate system^6^, ocean waves data are not available from the state-of-art global climate models (GCM). Using outputs of CMIP5/CMIP6 GCMs to force wave model, several ensembles of global wave climate projections are available for historical as well as for future periods, especially within the framework of the Coordinated Ocean Wave Climate Project (COWCLIP)^7^. In order to understand wave climate response to growing anthropogenic interference, we need ocean wave information for different forcing scenario. Anthropogenic greenhouse gases and aerosols are major external forcings to global climate change^8,9^. CMIP6 multi-model simulations^10^ provide scenarios for various external forcings under the Detection and Attribution Model Intercomparison Project (DAMIP)^11^. The goal of DAMIP is to attribute historically observed climate change to anthropogenic and natural causes, to derive observationally constrained projections of future climate change, and to improve understanding of the mechanisms by which particular forcings affect climate. Following the approach of Tier 1 experiments of DAMIP, we aim to create novel wave database corresponding to CMIP6 historical, hist-nat (natural-only), hist-GHG (greenhouse-only) and hist-aer (aerosol-only) experiments (described in Table 1) separately. Although few recent wave climate projections^12–14^ are available using CMIP6 forcings, these include all forcings together. The DECK (Diagnostic, Evaluation and Characterization of Klima) experiment^10^ within CMIP6 framework provides pre-industrial control simulation, which is representative of the period prior to the onset of large-scale industrialization, with 1850 being the reference year. The assumptions of the control simulation is that there are no changes in both naturally occurring forcings (e.g. volcanoes, orbital or solar changes) and human-induced forcings. Control simulation can thus be used to study the unforced internal variability of the climate system^11^. In addition to CMIP6 historical simulations, we aim to simulate wave conditions corresponding to pre-industrial control simulation using DECK results which will be useful to estimate the range of internal climate variability.

**Table 1:** CMIP6 experiments from MRI-ESM2.0 used in this study.

Experiment	Description	Duration
CTL	Pre-industrial control simulation time invariant representative of the year around 1850	60-year chunks (3)
ALL	CMIP6 historical simulation and SSP2-4.5 forced by evolving external forcing such as solar variability, volcanic aerosols, and changes in atmospheric composition (GHGs and aerosols) caused by human activities.	1960–2020
NAT	Natural-only historical simulations (solar irradiance, stratospheric/ volcanic aerosol)	1960–2020
GHG	Historical greenhouse-gas-only simulations forced by well-mixed greenhouse gas changes only	1960–2020
AER	Anthropogenic-aerosol-only historical simulations	1960–2020

Here, we provide 6-hourly time-series of wave parameters (e.g. significant wave height, mean wave period, mean wave direction) for 1960–2020 for different forcing scenario. This extensive digital wave database can be used by various scientific community and policymakers. A direct application is detection and attribution (D&A) studies of historical wave climate change to anthropogenic and natural causes, specifically relative contribution of anthrogopenic greenhouse and anthropogenic aerosols. This kind of D&A studies have never been attempted before for wave climate, to the best of our knowledge, owing to lack of dataset for different forcing scenario.

## Methods

### CMIP6 forcings

We derive surface winds (u and v components) at 3 hourly interval and sea ice area fraction at monthly interval from CMIP6 dataset (https://esgf-node.llnl.gov/projects/cmip6/) for historical (natural plus all anthropogenic, called ALL), greenhouse-only (GHG), natural-only (NAT), and aerosol-only forcing (AER) experiments. In CMIP6 dataset, there are many models which can provide surface winds at daily and monthly frequency for each of the above four experiments, but not at the 3 hourly frequency required to accurately reproduce synoptic-scale ocean wave generation. Therefore, we selected the MRI-ESM2.0, the Earth System Model (Version 2.0) by the Meteorological Research Institute^15^, as it provides 3 hourly surface wind (1.125° × 1.11209°~1.12148°), and monthly sea-ice area fraction for each of these four CMIP6 experiments: ALL, GHG, AER, NAT.

MRI-ESM2.0 is a global climate model consisting of four major component models: an atmospheric general circulation model (AGCM) with land processes, an ocean–sea-ice general circulation model, an aerosol model, and an atmospheric chemistry model. This climate model has been shown to display realistic reproduction of both mean climate and interannual variability^15^. Following a preindustrial spin-up, a preindustrial control experiment was conducted with MRI-ESM2.0 as well as a set of historical simulations from the 1850 to the present, which were driven by forcing based on observations. These experiments are conformed to the protocol specified by CMIP6.

**Fig. 1:**
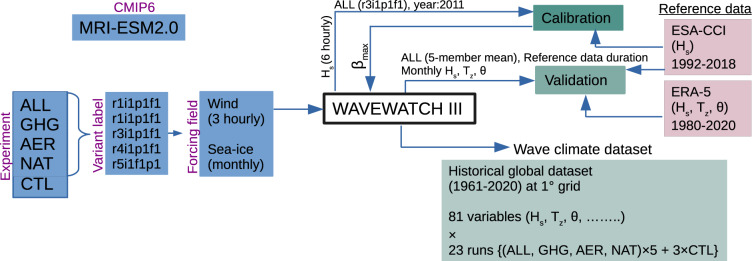
Schematic diagram of the methods used in this study to generate the wave climate dataset.

The ALL simulations generally end in 2014, but simulations are extended to 2020 with the Shared Economic Pathway (SSP) 2–4.5 scenario. SSP2-4.5^16^ is intermediate level of greenhouse gas emissions and its future aerosol and land use changes represent a broad range of SSP-based integrated assessment model projections^11^. Following DAMIP project, CMIP6 historical + SSP2-4.5 experiment is called “historical” here and covers the period 1960–2020. Five variants (r1i1p1f1, r2i1p1f1, r3i1p1f1, r4i1p1f1, r5i1p1f1) are available for the whole 1960–2020 period for each of the four experiments (Table 1 and Fig. 1). The different variant label defines the realization number (r), the initialization index (i), the physics index (p), and the forcing index (f); all being positive integer. For a given CMIP6 experiment (for example: Hist-GHG), the “ripf” identifier is used to uniquely identify each simulation of an ensemble of runs contributed by a single model (for more details, visit https://github.com/WCRP-CMIP/CMIP6_CVs). The realization index distinguishes among members of an ensemble of simulations that differ only in their initial conditions (e.g. initialized from different points in a control run). The initialization index is used to distinguish simulations performed under the same conditions but with different initialization procedures. The physics index indicates the physics version used by the model, and the forcing index is used to distinguish different variants of forcing applied. Since the control simulation (CTL) is considered as time invariant, available 200 years of MRI-ESM2.0 CTL data is divided into 3 sub-periods of 60 years which is the length of historical simulations.

### Wave modelling

WAVEWATCH III version 7.14 (The WAVEWATCH-III Development Group, 2019) was implemented over a near-global grid (78° S to 80° N) with a spatial resolution of 1.0° and using the GEBCO ocean bathymetry. The wave spectrum is discretized in 24 directions (15° directional resolution), and 36 exponentially spaced frequencies from 0.034 to 0.95 Hz, with a 1.1 increment factor from one frequency to the next. The maximum global time step, the maximum Courant–Friedrichs–Lewy (CFL) time steps for advection (x-y) and refraction (k-theta), and the minimum source term time step are set as 1800s, 300 s, 300 s, and 10 s, respectively. The wind input and wave dissipation source terms are from ST4 parameterization described in Ardhuin *et al*.^17^, with adjustments described in Alday *et al*.^18^. Non-linear wave interactions are modelled using the Discrete Interaction Approximation (DIA)^19^.

**Fig. 2:**
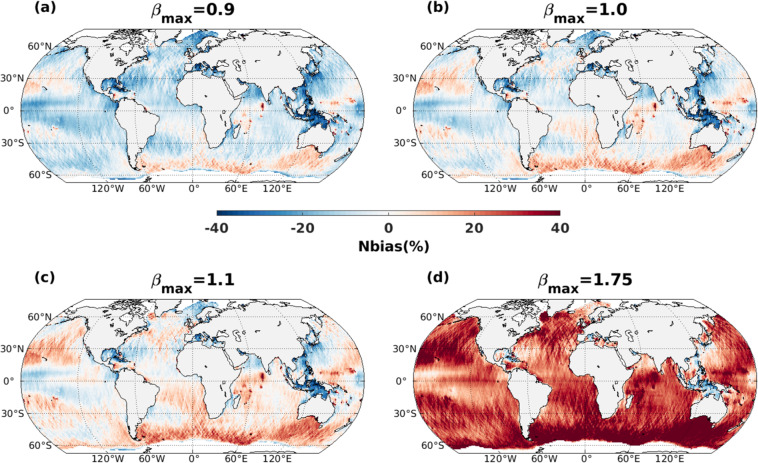
Normalized bias (%) between CMIP6_WW3 SWH from a single member of ALL simulations (r3i1p1f1) and altimeter SWH, for four *β*_*max*_ values, averaged over the year 2011.

Here, we adjusted the *β*_*max*_ parameter, which is a non-dimensional wind-wave growth coefficient that has been used as a tuning parameter to calibrate spectral wave models at global scale for wind strength biases^20^. Other specifications for model parameterizations are from test T475 from the WW3 hindcast implemented at Ifremer^18^. Model calibration and performance analysis are done by comparing the significant wave heights (SWH) obtained with the historical-ALL simulations and SWH from the inter-calibrated and denoised multi-mission satellite altimeter dataset (L4 product, version 1.1^21^) provided by the European Space Agency Climate Change Initiative (ESA-CCI)^22^ (Fig. 1). For model calibration, we looked at the systematic error statistics (bias and normalized bias: Nbias) computed over the full year of 2011 (same as Alday *et al*.^18^) using *β*_*max*_ values increasing from 0.9 to 1.75 (Table 2). The calibration analysis is based on a single year (enough to capture seasonal variability) in order to deal with computational demand. In this calibration analysis, the observation is 1 Hz along-track altimeter data and 6-hourly model outputs are interpolated at the position and time of altimeter measurements. First, the error metrics are computed for a single member from ALL simulations. We start with *β*_*max*_ = 1.75, as used by the reference study^18^, which displays high positive bias (not shown) and Nbias (Fig. 2d). After reducing *β*_*max*_ around 1, lower Nbias values are found. The area-averaged (60° S to 60° N) statistical values presented in Table 2 show the lowest bias (−0.04 m) and Nbias (−1.82%), when *β*_*max*_ = 1. These global average values are calculated after removing the outliers, from the spatial map, which are defined as values more than three scaled median absolute deviations from the median.

**Table 2:** Absolute and relative systematic error metrics for SWH (global averaged 60°S to 60°N) for the *β*_*max*_ sensitivity runs using ALL simulation (r3i1p1f1).

*β*_*max*_	0.9	1.0	1.1	1.75
Bias (m)	−0.16	−0.04	0.06	0.66
Nbias (%)	−6.81	−1.82	2.87	27.28

Following *β*_*max*_ = 1.0, all the members are shown to produce low bias (not shown here). Additionally, the standard deviations (of bias and Nbias) among the members are quite low. Here we acknowledge that the CMIP6 wind-forcing resolution is not adequate to reproduce the precise timing of storms^23^. Hence the random error metrics (scatter index: SI) using 6-hourly outputs are usually large (around 40%), even though the model captures the long-term climate well. After the *β*_*max*_ = 1.0 chosen with this calibration, the model setting is retained for the entire simulation. Thereafter, the model simulations are validated through Nbias, SI, SC (spatial correlation), NSD (normalized standard deviation) and CRMSD (centered root mean squared difference) metrics using monthly mean values for the entire reference data duration. The statistical parameters, used in this study, are defined as follows:

1 $$bias=\frac{\sum \left(M-R\right)}{count}$$

2 $$Nbias=\frac{\sum \left(M-R\right)}{\sum R}$$

3 $$SI=\sqrt{\frac{\sum {\left[\left(M-\bar{M}\right)-\left(R-\bar{R}\right)\right]}^{2}}{\sum {R}^{2}}}$$

4 $$SC=\frac{{\sum }_{x}\left(MR-\bar{M}\bar{R}\right)}{{\sum }_{x}\left({M}^{2}-{\bar{M}}^{2}\right)\left({R}^{2}-{\bar{R}}^{2}\right)}$$

5 $$NSD=\frac{{\sigma }_{M}}{{\sigma }_{R}}$$

6 $$CRMSD=\sqrt{\frac{\sum {\left[\left(M-\bar{M}\right)-\left(R-\bar{R}\right.\right]}^{2}}{count}}$$

where M and R are significant wave heights from model simulation and reference data, respectively. And *σ* is standard deviation.

## Data Records

The full global wave climate dataset^24^ can be accessed at 10.12770/0983962b-4acc-4f8f-9484-e2195029b87b.

The dataset consists of 6 hourly wave parameters (81 variables including significant wave height, mean wave periods, wave mean direction for total wave energy and partitions, among others), which are detailed in Supplementary Table 1, for 5 variants for each of the four CMIP6 historical experiments (ALL, NAT, GHG, AER) and three sets for CTL experiments (total = 23 runs) covering 60 years (1961–2020).

The 6-hourly outputs are saved into monthly files, so we have 12 × 60 × 23 netcdf files.

The hierarchy for folders is set as: GLOB1D_ exp_id/ 0r/year/.

The gridded outputs are in the folder /FIELD_NC;and the filenames are defined as: LOPS_WW3-glob-1d_YearMonth.nc,where Exp_id is the name of CMIP6 experiments, ALL, NAT, GHG, AER, CTLand 0r is the realization number of the variants (for example: 01 for r1i1p1f1, 02 for r2i1p1f1, and so on).

LOPS_WW3-glob-1d is WW3 global hindcast produced at LOPS for global ocean at 1° resolution.

The folders other than /FIELD_NC are:

“data/“ folder which has all the configuration files including bottom topography, namelist, etc.

“work/“ folder which has preprocessing file descrption used for each run.

“output/“ folder having output field description and others.

“SPEC_NC/“ folder having spectral energy information at the points listed in “/data/points_glob-1d.txt”.

## Technical Validation

**Fig. 3:**
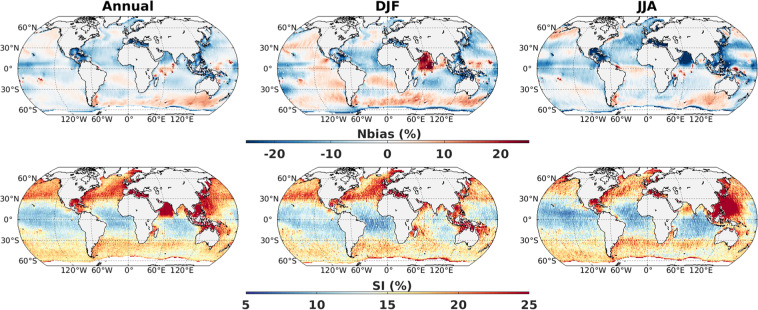
Normalized bias (%) and scatter index (%) of SWH from CMIP6_WW3 ALL simulations (5 member mean) with reference to altimeter during 1992–2018 using monthly mean data. Left columns are considering all months, middle columns are for December-January-February and right columns are for June-July-August.

In order to use this dataset for future wave climate research, we evaluate the skill of our model simulations (ensemble mean of ALL) using various metrics with reference to ESA-CCI altimeter^22^ and ERA5^25^ dataset. The skill of the wave database to capture the mean state is assessed through Nbias and SI maps from monthly mean data for annual and seasonal scales. At annual scale, the Nbias values are typically within 10% (Fig. 3), and SI values are typically within 15%, with values higher than 15% mostly located in high latitude, coastal and enclosed seas. Overall, it represents good agreement with altimeter observations with global averaged values −3% and 17% for Nbias and SI, respectively. The Nbias values are significantly lower than the ones obtained for the calibration of the model (shown on Fig. 2), as we used monthly mean and ensemble mean of 5 members. In DJF and JJA seasons, the Nbias and SI values are of similar ranges, but with regional variations (Fig. 3). Exception is the Arabian Sea, which shows overestimation in DJF season and underestimation in JJA season by WW3 simulation compared to altimeter observation. It seems that MRI-ESM2.0 has difficulties in resolving the South Asian Monsoon accurately. In addition, few localized abnormalously high bias values are scattered over tropical Indian Ocean which might occur due to unresolved sub-grid scale physics by our WW3 set-up with 1.0° horizontal resolution. Apparently, the current model discretization is coarse to capture island (Hawaii, Seychelles, Maldives, Chagos) blocking effect. It is noteworthy to mention that altimeter SWH measurements at low sea states (<0.75 m) is usually less accurate and noisier^26^, which might also be the reason for high SI values in coastal areas. We see high SI values over the regions with strong current, specially western boundary currents. In this context, Alday *et al*.^18^ reported reduction in scatter index for the regions with stronger current (such as the Gulf stream, the Kuroshio, the Agulhas, etc.) when current is added as forcing to wave model. Unfortunately, MRI-ESM2.0 does not provide data for ocean currents. Note that, although MRI-ESM2.0 has improved over the previous versions and demonstrates realistic aspects of many climate components, still it has drawbacks in terms of double intertropical convergence zone (ITCZ) bias, cold biases in the Southern Hemisphere (SH) surface air temperature, among others^15^. The inconsistency between model and observation can arise due to combination of multiple factors, including forcing field, parameterization, model discretization, and altimeter measurement errors. Here, model discretizations are a balance between the computational cost and the level of accuracy. The 60 years of hindcast at 6-hourly interval for 5 CMIP6 experiments, each having 5 variants, already demands a large computation storage.

**Fig. 4:**
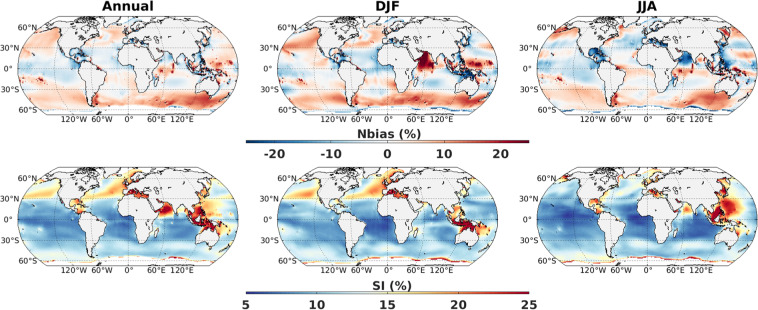
Normalized bias (%) and scatter index (%) of SWH from CMIP6_WW3 ALL simulations (5 member mean) with reference to ERA5 during 1980–2020 using monthly mean data. Left columns are considering all months, middle columns are for December-January-February and right columns are for June-July-August.

A slightly stronger Nbias and reduced SI values are seen when the model simulations are compared with ERA5 reanalysis (Fig. 4). Interestingly, spatial structure (positive and negative errors) and seasonal variation of Nbias pattern are similar for both reference data set (ESA-CCI altimeter and ERA5), which is partly due to the assimilation of altimeter data in ERA5. More specifically, estimates of Nbias computed with ERA5 show higher overestimation over the mid-latitudes and lesser underestimation over the intertropical regions compared to Nbias computed with altimeter, in agreement with the known differences between altimeter and ERA5^22^. The lower scattered index estimates can be explained by smoother ERA5 values compared to altimeter observations. The global averaged values are 2% and 12% for Nbias and SI, respectively.

It is important to acknowledge that differences in altimeter data processing methods may induce significant differences between multi-mission altimeter products^27,28^. Moreover, different global wave hindcasts and/or wave reanalysis often show disparate results regarding climatology, variability; as evidenced by Morim *et al*.^29^ and Erikson *et al*.^30^. As a consequence, the calibration and validation results may potentially vary depending on the selected reference dataset (observations or model). Since it is not the objective of this paper to compare different altimeter and reanalysis products, the reader could to refer to Morim *et al*.^29^ and Erikson *et al*.^30^ for comparative assessments of reference altimeter and model datasets.

As expected, higher error is seen for monthly maxima of SWH than for monthly mean values when compared with altimeter (Figure not shown). Model overestimates almost everywhere with larger values over the mid-latitudes of both hemisphere, and global averaged values are around 20%. The spatial pattern of SI map bears resemblance to that of monthly mean values (Fig. 3), but with amplified magnitude. As acknowledged in Young and Ribal^31^ and Timmermans *et al*.^27^, differences in extremes between products are more likely to be higher than in mean values. These large errors can be partially explained by the low revisit time period of altimeter missions, causing significant undersampling errors in multi-mission altimeter products^32^, which poorly resolve the extreme storm events.

**Fig. 5:**
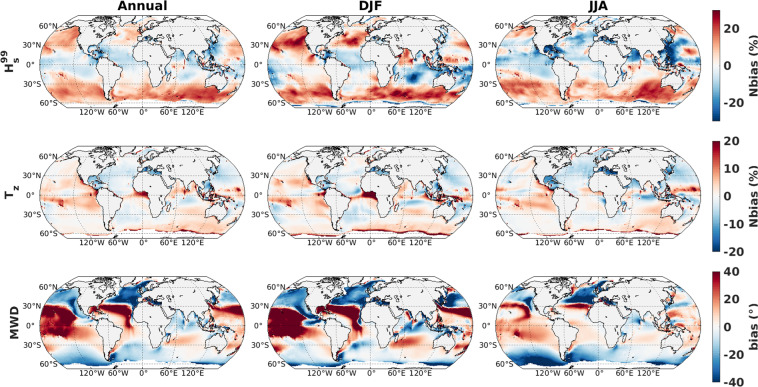
Normalized bias (%) of 99th percentile SWH $\left({H}_{s}^{99}\right)$ and mean zero-crossing wave period (*T*_*z*_); and bias of mean wave direction (MWD) from CMIP6_WW3 ALL simulations (5 member mean) with reference to ERA5 during 1980–2020 using monthly data. Left columns are considering all months, middle columns are for December-January-February and right columns are for June-July-August.

Given the noisiness of maximum wave heights, monthly 99th percentile SWHs (${H}_{s}^{99}$) are also validated with reference to ERA-5 wave height (Fig. 5). The spatial pattern of Nbias for ${H}_{s}^{99}$ shows resemblance to that of monthly mean values with higher overestimation in the storm track regions (high latitudes). Notable negative biases prevail over the Arabian Sea and east China sea during summer monsoon season. Contrasting relative difference for Arabian Sea between the two seasons is visible just in the case of monthly mean values. In case of wave period (T_*z*_), the Nbias values are within 10(%), except for east tropical Atlantic. However, limited overestimation is evident along the tropical latitudes of both hemispheres, in the so-called swell pools^33^. The mean wave direction from model shows positive (clockwise) biases along the tropical band, and slightly lower negative (anticlockwise) biases over the high-latitudes. The clockwise rotation can be explained by strong westerly component along the strom belt and the anticlockwise rotation by strong southerly component associated with strong swells from Southern Ocean; and similar pattern is reported by Lemos *et al*.^34^ when comparing with ERA5 dataset. Overall, our model simulations can reproduce the major spatial patterns and seasonal variations as in ERA5. The global average values for normalized bias for ${H}_{s}^{99}$, T_*z*_ and bias for MWD (using ERA5 as reference) are 3.07(%), 2.2(%), and 0.9 (°), respectively (Table 3). In addition to Nbias and SI, metrics such as SC, NSD, and CRMSD are also provided; as in COWCLIP framework^23^. The SC values are close to unity, while NSD values are 05~0.9. The CRMSD values are 1.04 m, 0.48 s, 35.01° for ${H}_{s}^{99}$, T_*z*_, and MWD, respectively. Moreover, these metrics are comparable with Song *et al*.^12^.

**Table 3:** Global averaged error metrics for CMIP6_WW3 ALL simulations (5-member mean) for reference data period (ESA-CCI :1992–2018 and ERA5: 1980–2020).

Reference	SC	NSD	CRMSD	Nbias%	SI%
ESA-CCI_*H*_*s*_ (m)	0.99	0.61	0.45	−3.1	16.8
ESA-CCI $\_{H}_{s}^{max}$ (m)	0.99	0.69	1.10	24.8	26.2
ERA5_*H*_*s*_ (m)	0.99	0.66	0.33	2.4	12.6
ERA5 $\_{H}_{s}^{99}$ (m)	0.99	0.89	1.04	3.1	20.5
ERA5_*T*_*z*_ (s)	0.95	0.62	0.48	2.2	6.8
ERA5_*MWD* (°)	0.96	0.52	35.01	0.9	N/A

Note that for MWD, the absolute bias value is provided (in°), instead of the normalized bias (%).

**Fig. 6:**
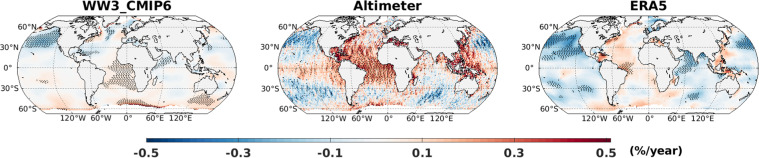
Linear trend (%) expressed as percentage change per year during 1992–2018 for SWH from CMIP6_WW3 ALL simulations, ESA-CCI altimeter, and ERA5 dataset.

To assess multi-annual variability, comparison of linear trend in annual mean wave height is presented in Fig. 6. Generally, the percentage change per year among three datasets agree on spatial distribution and sign of trends. Quantitatively, the range of the trends are also in agreements, although the altimeter trends are slightly stronger in certain regions (e.g. tropical Atlantic). Strong negative trends in the North Pacific is clearly consistent among the datasets. Other regions of consistent trends include Atlantic and eastern Pacific sector of Southern Ocean and tropical Atlantic. Nevertheless, certain differences in trends among three dataset are evident over Arabian Sea and mid-latitudes of south Pacific.

**Fig. 7:**
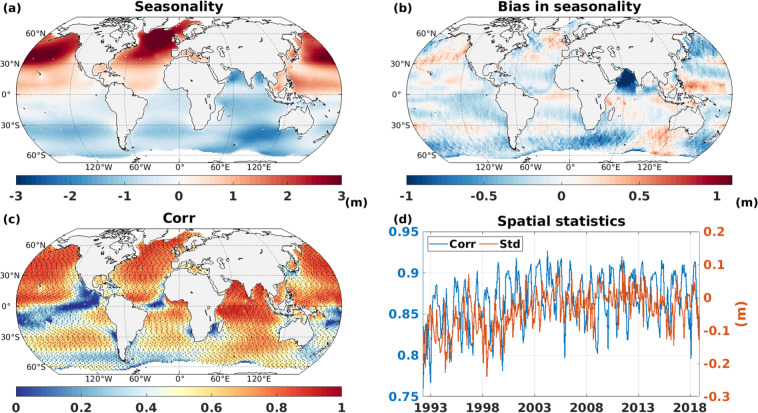
(**a**) Seasonality (January minus July) of modeled SWH (m) from ALL simulations, (**b**) Difference between ALL simulations and altimeter seasonality (m), (**c**) Time correlation (Pearson correlation coefficient) between ALL simulation and altimeter SWH with black dots showing statistical significance at 5% level, (**d**) spatial correlation coefficient (blue) and difference in spatial standard deviation (red) between ALL simulation and altimeter SWH (m) during 1992–2018.

Furthermore, we validate the ability of CMIP6-forced wave model to represent seasonal cycle. The modeled seasonality (difference between January and July wave height) is strongest in Northern Hemisphere (NH) extra-tropical storm belt with lower values over tropics (Fig. 7a). In SH, it is strongest over Indian Ocean sector of Southern Ocean, which is in agreement with Hemer and Trenham^35^. Looking at the bias in seasonality (Fig. 7b), it is encouraging that difference of seasonal amplitude between CMIP6 forced simulation and altimeter is generally lower than 0.5 m, except for the Arabian Sea and certain sectors of the Southern Ocean, which coincides with regions with strong bias (Fig. 3). Overall, the reduced agreement in Southern Ocean can be because of combination of factors, such as presence of circumpolar current, presence of both sea-ice and icebergs, the parameterizations of wave-ice interactions. In a quantitative view, difference in seasonal amplitude is reasonable with a low global mean value (−0.12 m). The temporal correlation map (using monthly mean values) reveal statistically significant relation almost everywhere in global ocean, with higher values (>0.7) in NH (Fig. 7c). Surprisingly, low to no correlation is seen over narrow tropical band and over a narrow latitudinal band close to Southern Ocean. These regions having low correlation coincides with regions having low seasonal or intra-annual variability (Fig. 7a). The spatial patterns of simulated dataset correlated well with the spatial pattern of altimeter observation over the years (blue curve on Fig. 7d), always higher than 0.75 except for the year 1992, probably because of altimeter undersampling issue^32^. To further evaluate spatial variability, difference in spatial standard deviation between WW3 simulation and altimeter is presented (red curve on Fig. 7d). The spatial variance of simulated dataset are generally within the same range as the observation, to be more specific, model variance is slightly stronger than observation since the year 2000. To summarise, this WW3 simulations produce considerably realistic representation of the historical global wave climate.

**Fig. 8:**
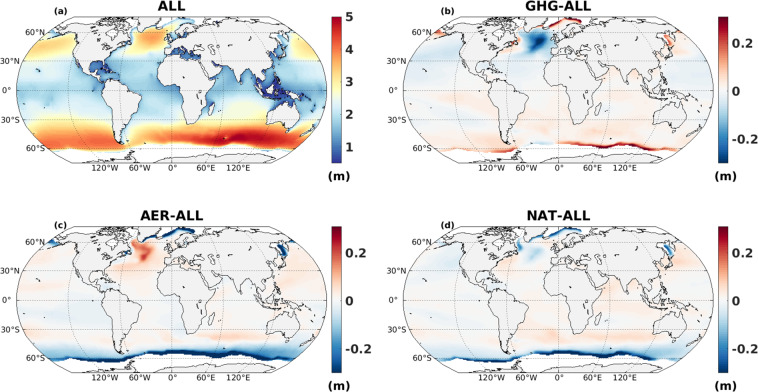
(**a**) Climatology of SWH (m) from ALL; and anomaly of climatology (with respect to ALL) for (**b**) GHG, (**c**) AER, and (**d**) NAT simulation during 1961–2020 (in m).

To present a brief overview of our simulated wave data, 60-year mean climatology for all the CMIP6 experiments: ALL, GHG, AER, and NAT are displayed in Fig. 8. Note that for GHG, AER and NAT scenarios, anomaly of climatology (60-year mean of GHG/AER/NAT - 60-year mean of ALL) with respect to ALL simulation is displayed. The ALL simulation clearly shows a global zonal distribution with larger values over extra-tropics and lower values in the tropics. Anomaly maps corresponding to different forcings differ substantially from each other and obviously from ALL forcing. In NAT, the anomaly amplitude is weaker than GHG and AER close to the Arctic circle. In the Southern Ocean, negative anomaly exists for AER and NAT in contrast to GHG positive anomaly. It is noteworthy that stronger anomalies are seen in the proximity of sea-ice. The contrasting behaviors among the difference forcing scenario is quite intriguing. However, the potential reason behind such pattern is not in the scope of current study but clearly warrants further research. Detailed attribution of the differences between datasets would be desirable, through understanding skill of the forcing obtained from climate model, although many uncertainty remains in the reference observations^27^. We acknowledge that, our wave product is using single-method modelling, so uncertainty arising from different global wave products should be considered when assessing coastal hazards/risk for extremes^36^. Our dataset will be of service to future research to clarify how external anthropogenic and natural forcing have influenced historical wave climate changes.

## Supplementary information

Supplementary Table 1

## Data Availability

The WAVEWATCH III setup files are available in the data link (https://data-dataref.ifremer.fr/ww3/GLOB1D_CMIP6/)^24^ for the purpose of replicating the data described in this paper. The “wavesetup.env” file can be found in the following repository of the above link: GLOB1D_ exp_id/ 0r/year/. Please refer to Data Records section for more description of the repository.
