# Supplementary material for: Historical global ocean wave data simulated with CMIP6 anthropogenic and natural forcings
Source: Sci Data. 2023 May 26;10:325. doi: 10.1038/s41597-023-02228-6 (PMC10220036; doi:10.1038/s41597-023-02228-6)
Supplement: Supplementary file 1 — Supplementary Table 1 [file 41597_2023_2228_MOESM1_ESM.pdf]

Historical global ocean wave data simulated with CMIP6 anthropogenic and natural forcings

Anindita Patra, Guillaume Dodet, and Mickael Accensi

Univ. Brest, CNRS, Ifremer, IRD, Laboratoire d'Océanographie Physique et Spatiale, Brest, France

†anindita.patra@ifremer.fr

List of Parameters from Wavewatch III output	
1.	name=MAPSTA<br>standard_name=status map<br>long_name="status map"<br>units="1"
2.	name=dpt<br>standard_name=depth<br>long_name="depth"<br>units="m"
3.	name=uwnd<br>standard_name=eastward_wind<br>long_name="eastward_wind"<br>units="m s-1"
4.	name=vwnd<br>standard_name=northward_wind<br>long_name="northward_wind"<br>units="m s-1"
5.	name=ice<br>standard_name=sea_ice_area_fraction<br>long_name="sea ice area fraction"<br>units="1"
6.	name=hs<br>standard_name=sea_surface_wave_significant_height<br>long_name="significant height of wind and swell waves"<br>units="m"
7.	name=lm<br>long_name="mean wave length"<br>units="m"
8.	name=t02<br>standard_name=sea_surface_wind_wave_mean_period_from_variance_spectral_densit

	y_second_frequency_moment<br>long_name="mean period T02"<br>units="s"
9.	name=t0m1<br>standard_name=sea_surface_wind_wave_mean_period_from_variance_spectral_density_inverse_frequency_moment<br>long_name="mean period T0m1"<br>units="s"
10.	name=t01<br>standard_name=sea_surface_wind_wave_mean_period_from_variance_spectral_density_first_frequency_moment<br>long_name="mean period T01"<br>units="s"
11.	name=fp<br>long_name="wave peak frequency"<br>units="s-1"
12.	name=dir<br>standard_name=sea_surface_wave_from_direction<br>long_name="wave mean direction"<br>units="degree"
13.	name=spr<br>standard_name=sea_surface_wave_directional_spread<br>long_name="directional spread"<br>units="degree"
14.	name=dp<br>standard_name=sea_surface_wave_peak_direction<br>long_name="peak direction"<br>units="degree"
15.	name=phs0<br>standard_name=sea_surface_wind_wave_significant_height<br>long_name="wave significant height partition 0"<br>units="m"
16.	name=phs1<br>standard_name=sea_surface_primary_swell_wave_significant_height<br>long_name="wave significant height partition 1"<br>units="m"
17.	name=phs2<br>standard_name=sea_surface_secondary_swell_wave_significant_height<br>long_name="wave significant height partition 2"<br>units="m"
18.	name=phs3<br>standard_name=sea_surface_tertiary_swell_wave_significant_height<br>long_name="wave significant height partition 3"<br>units="m"
19.	name=phs4<br>standard_name=sea_surface_swell_wave_significant_height<br>long_name="wave significant height partition 4"<br>units="m"
20.	name=phs5<br>standard_name=sea_surface_swell_wave_significant_height

	long_name="wave significant height partition 5"<br>units="m"
21.	name=ptp0<br><br>standard_name=sea_surface_wind_wave_period_at_variance_spectral_density_maxim<br>um<br>long_name="peak period partition 0"<br>units="s"
22.	name=ptp1<br><br>standard_name=sea_surface_primary_swell_wave_period_at_variance_spectral_densit<br>y_maximum<br>long_name="peak period partition 1"<br>units="s"
23.	name=ptp2<br><br>standard_name=sea_surface_secondary_swell_wave_period_at_variance_spectral_den<br>sity_maximum<br>long_name="peak period partition 2"<br>units="s"
24.	name=ptp3<br><br>standard_name=sea_surface_tertiary_swell_wave_period_at_variance_spectral_density<br>_maximum<br>long_name="peak period partition 3"<br>units="s"
25.	name=ptp4<br><br>standard_name=sea_surface_swell_wave_period_at_variance_spectral_density_maxim<br>um<br>long_name="peak period partition 4"<br>units="s"
26.	name=ptp5<br><br>standard_name=sea_surface_swell_wave_period_at_variance_spectral_density_maxim<br>um<br>long_name="peak period partition 5"<br>units="s"
27.	name=plp0<br>long_name="peak wave length partition 0"<br>units="m"
28.	name=plp1<br>long_name="peak wave length partition 1"<br>units="m"
29.	name=plp2<br>long_name="peak wave length partition 2"<br>units="m"
30.	name=plp3<br>long_name="peak wave length partition 3"<br>units="m"

31.	name=plp4<br>long_name="peak wave length partition 4"<br>units="m"
32.	name=plp5<br>long_name="peak wave length partition 5"<br>units="m"
33.	name=pdir0<br>standard_name=sea_surface_wind_wave_from_direction<br>long_name="wave mean direction partition 0"<br>units="degree"
34.	name=pdir1<br>standard_name=sea_surface_primary_swell_wave_from_direction<br>long_name="wave mean direction partition 1"<br>units="degree"
35.	name=pdir2<br>standard_name=sea_surface_secondary_swell_wave_from_direction<br>long_name="wave mean direction partition 2"<br>units="degree"
36.	name=pdir3<br>standard_name=sea_surface_tertiary_swell_wave_from_direction<br>long_name="wave mean direction partition 3"<br>units="degree"
37.	name=pdir4<br>standard_name=sea_surface_swell_wave_from_direction<br>long_name="wave mean direction partition 4"<br>units="degree"
38.	name=pdir5<br>standard_name=sea_surface_swell_wave_from_direction<br>long_name="wave mean direction partition 5"<br>units="degree"
39.	name=pspr0<br>standard_name=sea_surface_wind_wave_diectional_spread<br>long_name="directional spread partition 0"<br>units="degree"
40.	name=pspr1<br>standard_name=sea_surface_primary_swell_wave_diectional_spread<br>long_name="directional spread partition 1"<br>units="degree"
41.	name=pspr2<br>standard_name=sea_surface_secondary_swell_wave_diectional_spread<br>long_name="directional spread partition 2"<br>units="degree"
42.	name=pspr3<br>standard_name=sea_surface_tertiary_swell_wave_diectional_spread<br>long_name="directional spread partition 3"<br>units="degree"
43.	name=pspr4<br>standard_name=sea_surface_swell_wave_diectional_spread<br>long_name="directional spread partition 4"<br>units="degree"

44.	name=pspr5<br>standard_name=sea_surface_swell_wave_diectional_spread<br>long_name="directional spread partition 5"<br>units="degree"
45.	name=pws0<br>long_name="wind sea fraction in partition 0"<br>units="1"
46.	name=pws1<br>long_name="wind sea fraction in partition 1"<br>units="1"
47.	name=pws2<br>long_name="wind sea fraction in partition 2"<br>units="1"
48.	name=pws3<br>long_name="wind sea fraction in partition 3"<br>units="1"
49.	name=pws4<br>long_name="wind sea fraction in partition 4"<br>units="1"
50.	name=pws5<br>long_name="wind sea fraction in partition 5"<br>units="1"
51.	name=pdp0<br>standard_name=sea_surface_wind_wave_from_direction_at_variance_spectral_density_maximum<br>long_name="peak direction partition 0"<br>units="degree"
52.	name=pdp1<br>standard_name=sea_surface_primary_swell_wave_from_direction_at_variance_spectral_density_maximum<br>long_name="peak direction partition 1"<br>units="degree"
53.	name=pdp2<br>standard_name=sea_surface_secondary_swell_wave_from_direction_at_variance_spectral_density_maximum<br>long_name="peak direction partition 2"<br>units="degree"
54.	name=pdp3<br>standard_name=sea_surface_tertiary_swell_wave_from_direction_at_variance_spectral_density_maximum<br>long_name="peak direction partition 3"<br>units="degree"
55.	name=pdp4<br>standard_name=sea_surface_swell_wave_from_direction_at_variance_spectral_density_maximum

	long_name="peak direction partition 4"<br>units="degree"
56.	name=pdp5<br><br>standard_name=sea_surface_swell_wave_from_direction_at_variance_spectral_density_maximum<br>long_name="peak direction partition 5"<br>units="degree"
57.	name=tws<br>long_name="wind sea fraction"<br>units="1"
58.	name=uust<br>long_name="eastward friction velocity"<br>units="m s-1"
59.	name=vust<br>long_name="northward friction velocity"<br>units="m s-1"
60.	name=cha<br><br>standard_name=charnock_coefficient_for_surface_roughness_length_for_momentum_in_air<br>long_name="charnock coefficient for surface roughness length for momentum in air"<br>units="1"
61.	name=cge<br>long_name="wave energy flux"<br>units="kW m-1"
62.	name=faw<br>standard_name=wind_mixing_energy_flux_into_sea_water<br>long_name="wind to wave energy flux"<br>units="W m-2"
63.	name=utaw<br>long_name="eastward wave supported wind stress"<br>units="m2 s-2"
64.	name=vtaw<br>long_name="northward wave supported wind stress"<br>units="m2 s-2"
65.	name=utwa<br>long_name="eastward wave to wind stress"<br>units="m2 s-2"
66.	name=vtwa<br>long_name="northward wave to wind stress"<br>units="m2 s-2"
67.	name=wcc<br>long_name="whitecap coverage"<br>units="1"
68.	name=utwo<br>long_name="eastward wave to ocean stress"<br>units="m2 s-2"
69.	name=vtwo

	long_name="northward wave to ocean stress"<br>units="m2 s-2"
70.	name=foc<br>long_name="wave to ocean energy flux"<br>units="W m-2"
71.	name=utus<br>long_name="eastward stokes transport"<br>units="m2 s-1"
72.	name=vtus<br>long_name="northward stokes transport"<br>units="m2 s-1"
73.	name=uuss<br>standard_name=sea_surface_wave_stokes_drift_eastward_velocity<br>long_name="eastward surface stokes drift"<br>units="m s-1"
74.	name=vuss<br>standard_name=sea_surface_wave_stokes_drift_northward_velocity<br>long_name="northward surface stokes drift"<br>units="m s-1"
75.	name=uabr<br>long_name="rms of bottom displacement amplitude zonal"<br>units="m"
76.	name=vabr<br>long_name="rms of bottom displacement amplitude meridional"<br>units="m"
77.	name=uubr<br>long_name="rms of bottom velocity amplitude zonal"<br>units="m s-1"
78.	name=vubr<br>long_name="rms of bottom velocity amplitude meridional"<br>units="m s-1"
79.	name=mssu<br>standard_name=sea_surface_wave_mean_square_upwave_slope<br>long_name="downwave mean square slope"<br>units="1"
80.	name=mssc<br>standard_name=sea_surface_wave_mean_square_crosswave_slope<br>long_name="crosswave mean square slope"<br>units="1"
81.	name=mssd<br>standard_name=sea_surface_mean_square_upwave_slope_direction<br>long_name="u direction for mss"<br>units="degree"
